# Fruit, Vegetable, and Fibre Intake among Finnish Preschoolers in Relation to Preschool-Level Facilitators and Barriers to Healthy Nutrition

**DOI:** 10.3390/nu11071458

**Published:** 2019-06-27

**Authors:** Reetta Lehto, Carola Ray, Liisa Korkalo, Henna Vepsäläinen, Kaija Nissinen, Leena Koivusilta, Eva Roos, Maijaliisa Erkkola

**Affiliations:** 1 Folkhälsan Research Center, Topeliuksenkatu 20, 00250 Helsinki, Finland; 2 Department of Food and Nutrition, University of Helsinki, 00014 Helsinki, Finland; 3 School of Food and Agriculture, Seinäjoki University of Applied Sciences, 60101 Seinäjoki, Finland; 4 Department of Social Research, University of Turku, 20014 Turku, Finland; 5 Department of Public Health, Clinicum, University of Helsinki, 00014 Helsinki, Finland

**Keywords:** childcare, dietary intake, children, environmental influences, manager

## Abstract

Preschool is a major factor affecting food consumption among young children in Finland, given that most preschoolers eat three meals a day in that setting. Thus, it is important to recognise the determinants of dietary intake at preschool. The aim of this study was to examine food-related factors at the preschool and manager level, and their association with the dietary intake of children in childcare. The study was a part of the cross-sectional DAGIS survey conducted in 2015 to 2016 in Finland. The managers of 58 preschools filled in a questionnaire related to food and nutrition at their preschools. Preschool personnel kept food records for the children (*n* = 585) on two preschool days. Multilevel linear and logistic regression analyses were conducted with age, gender, and municipality as covariates, preschool-level factors as independent variables, and children’s vegetable (g/day) and fruit (yes vs. no) consumption and fibre intake (g/MJ) as outcome variables. Having many written food policies in the preschool was associated with a higher intake of vegetables (*p* = 0.01) and fibre (*p* = 0.03) among the children. Having at least two out of three cooperation-related challenges with the catering service was associated with a higher intake of fibre (*p* = 0.03) and lower odds of eating fruit (*p* = 0.01). Factors that are relatively distal from meal situations may have an effect, and should be taken into account in the promotion of healthy eating at preschool, but more studies are needed.

## 1. Introduction

In Finland and other Western countries, the majority of children under school age attend formal childcare [1], from here on referred to as preschools, where most of them eat several meals a day. Thus, food intake at preschools constitutes a significant proportion of the children’s total dietary intake, and may have an impact on the formation of eating habits [2]. Recognising the factors that contribute to dietary intake at preschool would be beneficial given the increased prevalence of obesity among children [3] and the consequent need to promote a healthy diet at a young age.

According to socio-ecological models, the environment (in addition to personal characteristics) affects an individual’s health behaviour on several levels [4] representing environmental factors ranging from the immediate surroundings, such as home, to more distal levels, such as the organisational and societal level. Each level has its various physical, social, and political factors. Preschool could be seen as one level that affects children’s health behaviour. There has been an increasing amount of research on the preschool environment and children’s food intake in recent years. Most of the factors under study relate directly to meal times, such as the (un)healthiness of the food served [5,6,7] and, to some extent, the feeding practices of the personnel [8,9,10,11]. Less attention has been given to other preschool-level factors that are more distal from meal times. These factors could be characterised as political (e.g., preschool-level rules and policies concerning food), sociocultural (preschool-level practices including the personnel’s knowledge about nutrition), physical (such as whether the food is cooked on the premises), and managerial (e.g., the manager’s perceptions and attitudes).

Associations have been found linking food-related policies and regulations with the food and beverages served in childcare establishments [12], as well as with children’s dietary intake [13]. It appears that, in the US, healthier food is served to children in childcare centres that participate in the Child and Adult Care Food Program (CACFP) [14,15], which are more heavily regulated by the state and receive reimbursement for serving healthier food. There has been some qualitative research on preschool managers’ views about the barriers to and facilitators of healthy food intake and healthy energy-balance-related behaviour at preschool, but these studies did not examine any associations with the children’s food intake [16]. According to Van de Kolk [16], Dutch preschool managers mentioned physical (e.g., food availability), sociocultural (e.g., the personnel’s feeding practices), economic (e.g., food costs), and political (existing policies and guidelines) factors as both facilitators of and barriers inhibiting healthy eating among children. Studies in other settings also refer to the importance of the person in charge: In Canada, Olstad et al. [17] concluded in their study of managers in recreational sports settings that nutritional knowledge, attitudes, and perceptions shape their decisions and actions regarding the adoption and implementation of dietary guidelines.

Most preschools in Finland are municipal (public). All food is provided, and children are not allowed to bring their own food with them. Preschools provide three meals a day: Breakfast (often porridge), a warm meal at lunchtime, and an afternoon snack. There are national recommendations concerning dietary requirements and feeding practices [18,19], but these are not binding. The food is cooked either at the premises of municipal or private catering services (central kitchens) or in the preschool’s own kitchen (following the same menu as other municipal preschools). Either the municipality or the private catering service from which it buys its preschool meals is responsible for planning the menus and purchasing the food. Although the municipalities play a major role when it comes to food availability and mealtime practices, preschool managers may also influence food-related practices and policies.

Our aim in this study was to examine the association between preschool-level factors and the dietary intake of children during preschool hours. We focused on the following preschool factors: Food policies, the manager’s concern about the children’s consumption of fruit and vegetables, the manager’s perceived influence on the supply of fruit and vegetables, cooperation challenges with the catering service as perceived by the manager, the lack of resources, and kitchen type (whether the preschool kitchen is used for cooking or heating up food, or for distribution). The dietary factors we studied included the children’s fruit and vegetable consumption and their fibre intake.

## 2. Materials and Methods

This study is part of the cross-sectional survey “Increased Health and Wellbeing in Preschools” (DAGIS) conducted in Finnish preschools in 2015–2016 [20,21]. The aim of the whole survey was to examine health behaviour and stress regulation among preschool children aged between three and six years in relation to socioeconomic status and both the home and the preschool environments. The study was conducted in accordance with the Declaration of Helsinki, and the protocol was approved by the Ethics Committee of the University of Helsinki Ethical Review Board in the Humanities and Social and Behavioural Sciences in February 2015 (#6/2015). The cross-sectional survey was conducted in eight municipalities in Southern and Western Finland. A total of 169 municipal preschools were invited to participate. The eligibility criteria were: (a) The language of the preschool must be Finnish or Swedish; (b) it must be public (municipal), or the municipality should purchase early-education services from it; (c) it should function only during the daytime (excluding 24-hour preschools); (d) like all municipal preschools, it should charge income-dependent fees; e) it should have at least one group of approximately three-to-six-year-old children. Sixteen of the invited preschools were excluded because they did not meet the eligibility criteria. Of the remaining 153, 56% (86) agreed to participate. Families of the three-to-six-year-old children were invited to take part in the survey via the preschools. If the overall family-participation rate remained under 30%, the study was not conducted in that particular preschool. This was the case in 20 preschools, hence 66 were eligible to take part in the study. Of the 3592 families contacted in those preschools, 892 (27%) agreed to take part. Ultimately, we collected at least some research data from 864 children (24% of those invited), whom we consider the participants of the survey. The recruitment of the participants and the flow chart have been thoroughly described previously [21].

### 2.1. Preschool Factors

Preschool managers filled in a questionnaire covering policies, preschool-level practices, and their own views on factors related to the health behaviour of children at preschool. We received filled-in questionnaires from 53 preschool managers encompassing 58 preschools, as five managers filled in separate questionnaires for the two preschools for which they were responsible. We used seven manager-reported variables in the analyses, many of which were summed up from several questions/statements. The variables are grouped under the following headings: Policy factors, sociocultural factors, physical factors, and manager-related psychosocial factors. All the variables are presented in Table 1.

### 2.2. Policy Factors

The managers were asked if their preschool had written policies (preschool’s own or municipal/national) concerning 18 different food/nutrition-related matters, such as using food as a reward or punishment, and birthday treats (see Table 1). The policies were summed up and the preschools were divided into tertiles according to the number of policies in place (Table 2).

### 2.3. Sociocultural Factors

The first sociocultural factor represents food education at the preschool. This variable combines three questions: (i) Has the preschool offered its personnel in-service training on children’s nutrition during the last two years? (at least once vs. no); (ii) Have there been preschool theme weeks focusing on food/nutrition during the last two years? (at least once vs. no); (iii) Has the preschool ever used or does it currently use the Sapere method [22]? (yes/no). A positive response scored one point on each of these three actions, and the variable was categorised in three classes: 0, 1, and 2 or 3 points. The second sociocultural factor concerned challenges related to cooperation with the catering service. The managers were asked whether (i) a lack of cooperation with the catering-service personnel inhibited healthy nutrition, (ii) the limitations of the catering service inhibited healthy nutrition or food education, and (iii) communication between the preschool personnel and the catering personnel was fluent. The variable was categorised in three classes: 0, 1, and 2 or 3 barriers. The third sociocultural factor related to a lack of resources as a barrier to children’s healthy nutrition at preschool, broken down into three resource types. The managers reported separately on whether a lack of planning time, a lack of personnel, and a lack of materials inhibited healthy nutrition (yes/no). The responses were then classified as indicating either at least one of these barriers or none of them.

### 2.4. Manager-Related Psychosocial Factors

We considered two psychosocial factors. The first was the manager’s concern about the fruit and vegetable intake of the children, assessed from their responses to two questions. First, they were asked: “To what extent do you think there is generally a problem among three-six-year-old children concerning a low intake of vegetables/low intake of fruit?”. The responses were given on a five-point Likert scale (1–5) ranging from “not a problem” to “a very big problem,” summed and divided into three groups based on the distribution of the answers (Table 2). Second, they were asked if they had any influence in terms of the fruit and vegetable content of preschool meals. The question was asked separately for all meals. The answers were combined in a single dichotomous variable, since the same managers answered similarly to all questions (yes to all questions vs. no to all questions).

### 2.5. Physical Factors

The managers were asked what type of kitchen facilities the preschool had: (a) Cooking, (b) distribution, (c) heating, (d) other, what? and (e) there is no kitchen. The responses were categorised as cooking/heating vs. all other alternatives to distinguish the kitchens in which at least some cooking was done. The managers who responded other, what? (*n* = 3) were categorized in the right group based on their open answers following the question.

### 2.6. Children’s Dietary Intake

The children’s food intake while at preschool was measured using a two-day pre-coded food record that was kept by the personnel and covered the food and drink consumed as well as the amounts. Breakfast, lunch, and the afternoon snack had predefined templates grouping food commonly consumed at each meal to make the record-keeping easier. The personnel estimated portion sizes in household measures, or with the help of a validated Children’s Food Picture Book [23,24] that is specifically designed to facilitate the estimation of portion sizes among children. Five of the eight municipalities supplied complete recipes for the food obtained from the preschool catering service, one municipality supplied some recipes, and two did not give any. When no recipe was available, recipes from other catering services were used. The food-record data were entered into the dietary-calculation software AivoDiet, version 2.2.0.0 (Aivo Finland Oy, Turku, Finland) for the computation of the children’s food consumption and nutrient intake. The program uses the Finnish national food composition database, Fineli [25].

In this study, we used fruit consumption (yes vs. no), vegetable consumption (g/day), and fibre density (g/MJ) as the relevant dietary factors representing a healthy dietary intake. The Finnish child-nutrition recommendation for vegetables and fruit combined is a minimum of five handfuls (about 250 g), and for fibre density, 3 g/MJ a day [19]. A dichotomous variable was used for fruit consumption because a large proportion (37%) of the children did not eat any fruit during the two days of food record-keeping. Vegetable consumption included raw and cooked vegetables eaten as such, but not vegetables included in main dishes. Potatoes were not considered vegetables. We used the data from children who had eaten all the preschool meals (breakfast, lunch, and afternoon snack) on at least one of the two days when the food record was being kept. Both vegetable consumption per day and fibre density per day were calculated so that if the child had eaten the same meal (e.g., lunch) on both days, the mean intake was used. For example, if a child had eaten lunch and an afternoon snack on both days, but breakfast on only one day, the mean daily intake was calculated as the sum of the one breakfast and the mean intake of the two lunches and snacks.

### 2.7. Statistical Analyses

First, we checked the distributions of vegetable consumption and fibre density. Unlike fibre density, vegetable consumption was not normally distributed and thus we used square root transformation. We subjected the associations between the preschool-level factors and children’s dietary intake to multi-level linear and logistic regression analysis with preschool as the random effect: The manager was included in the models as a new random effect, but the result was not statistically significant. We used two models: A crude model with no adjustments and a model adjusting for the child’s age, gender, and municipality (as a dummy variable). Results of the linear regression analyses are expressed as beta coefficients (beta) and those of logistic regression analyses as odds ratios (OR). Furthermore, a 95% confidence interval (CI) is used with both. In one municipality, there were only three children who met the inclusion criteria of having three meals at preschool and they were all from the same preschool: We deleted these children from the analyses due to the lack of variation. IBM Statistics SPSS version 25.0 (SPSS Inc., Chicago, IL, USA) and statistical software Mplus version 7.4 [26] were used for the analyses.

## 3. Results

Table 2 shows the descriptive results for the preschool variables. Most managers did not report a lack of resources or cooperation challenges with the catering service. Four out of five indicated that they could not influence the content of fruit and vegetables in the preschool meals, and most of them reported having less than half of the food policies. Table 3 gives the characteristics of the children and the preschool managers. All preschool managers were women. Less than half of the children were girls and children’s mean age was 4.7 years. Table 4 describes the dietary intake of the children. Food records were kept for 822 children, but the 586 children (72% of them) who had eaten all three meals at preschool on at least one of the days the food record was kept constitute the participants of this study. These children did not differ from the others in terms of age, gender, or socioeconomic background. Of them, 63% had eaten fruit, and on average, they consumed 39 g of raw and cooked vegetables and 9 g of dietary fibre during a preschool day.

Having at least 10 of the 18 food policies in place (i.e., being in the highest tertile of the number of food policies), compared to less than five (i.e., the lowest tertile), was associated with higher vegetable consumption (beta coefficient 0.89, 95% CI 0.20–1.58) and a higher fibre intake (beta coefficient 0.24, 95% CI 0.02–0.46) among the children (Table 5). Among the sociocultural factors, associations were found regarding cooperation-related challenges with the catering service: Having at least two of the three types of challenge, compared to having none, was associated with a higher fibre intake among the children (beta coefficient 0.22, 95% CI 0.03–0.42) and a lower likelihood of eating fruit at preschool (OR 0.28, 95% CI 0.11–0.76). Neither the other sociocultural factors, such as food education, nor manager-related psychosocial factors, such as perceived influence over fruit and vegetable supply, were associated with the children’s intake of fruit, vegetables, or fibre.

## 4. Discussion

We examined the extent to which certain preschool-level factors other than food availability and mealtime practices are associated with the intake of fruit, vegetables, and fibre among three- to six-year-old Finnish preschoolers. A larger number of food-related policies was associated with a higher consumption of vegetables and a higher intake of fibre. A higher number of manager-perceived challenges related to cooperation with the catering service was associated with lower odds of children eating fruit, and also with a higher fibre intake. However, kitchen type, food education, and a lack of resources were not associated with dietary intake. Apart from food policies, the preschool-level factors we used have seldom been studied, and to our knowledge, this is the first study to report on their associations with the food intake of children.

Some previous studies have reported an association between food policies and children’s dietary intake [12,13]. Himberg-Sundet et al. found that having their own written guidelines concerning the food and beverages that preschools offer to children was associated with a higher consumption of vegetables. According to the managers, these guidelines were developed largely in line with national recommendations. In our study, too, having written policies (its own or municipal/national) was associated with higher vegetable consumption and fibre intake in the preschool. Treating all policies as equal is misleading, however, given the variation in their type, content, and the extent to which they are enforced [27]. Some countries have laws concerning specific foods and beverages used in childcare establishments, and law changes have been found effective in changing the supply of beverages, for example [12]. National recommendations on preschool food and feeding practices are not binding in Finland, however, and compliance is not monitored nationally. In addition, given that neither the managers nor the preschools decide what kind of food and beverages will be served to children in Finnish preschools, most of the policies we studied concerned food education and feeding practices. In other studies that have examined food policies, the policies included have mostly concerned foods that should or should not be served to the children [12,13,27]. It would be useful in future analyses to investigate any association between the content of the policies and/or the extent to which the policies are enforced and the children’s dietary intake.

Compliance with national or municipal policies may vary depending on the manager’s or the personnel’s own interests in promoting healthy nutrition among children [17], and on the environmental context, resources, or social influences [28]. As we do not know the content of the policies nor the extent of compliance with them in preschools, we cannot hypothesise how they contribute to higher vegetable consumption and fibre intake. As cross-sectional associations, the associations which were found might also not be causal.

Perceived challenges related to cooperation with the catering service were related to both lower odds of eating fruit and a higher intake of fibre. To our knowledge, this is the first study to examine and find associations between cooperation-related conflict between preschools and catering services with children’s food intake. Byrd-Williams et al. [29] investigated perceived barriers to healthy nutrition at preschool among managers and other personnel and found that, to some extent, the limitations of the food-service provider and a lack of support from its personnel constituted such barriers. Moreover, the preschool personnel reported more barriers than the managers did [29]. The managers mentioned a similar number of barriers as our informants did [30].

The surprising finding of an association between a higher level of challenges related to the catering service and a higher intake of fibre could be attributable to the sources of dietary fibre. Nissinen et al. [30] examined nutrient intake and sources at preschool and at home among participants of the DAGIS survey, and found that the major source of dietary fibre at preschool was cereals (~64%), whereas only 19% came from fruit and vegetables and vegetable dishes. In fact, crispbread, which is commonly available as part of every preschool lunch, accounted for 21% of dietary-fibre intake: Eating a lot of crispbread could mean that other parts of the meal are not eaten, and it is usually discouraged. It would be worth finding out whether there are other differences in food intake, concerning the main dish for example, depending on the perceived challenges with the catering service. Given that challenges were also associated with a lower likelihood of children eating fruit, further investigation focusing on cooperation between the preschool and the catering service would be warranted.

Our examination of environmental factors and their associations with children’s food intake was limited to one socioecological level (preschool) and it examined only single associations at a time. As food intake is affected by a web of factors, it would be interesting in future studies to focus on interactions within and between levels, as well as on mediation from a more distal level to food intake via factors in the immediate surroundings. In addition, as cross-sectional studies cannot verify causality, longitudinal studies, and especially intervention studies, on preschool environments’ effects on children’s food intake are warranted. Finally, we examined a limited number of preschool-level factors, and acknowledge the need to investigate numerous political, economic, sociocultural, and physical aspects, including the personal characteristics and interests of managers.

The major strength of our study is its focus on hitherto rather neglected associations between a variety of preschool-level factors and children’s fruit and vegetable consumption and fibre intake at preschool. Another strength is the rigorous data on food intake gathered from the children by means of food records kept by the personnel and recipes obtained from catering services. Having conducted a validation study, Nissinen et al. [24] found that the preschool personnel’s assessments of children’s portion sizes from the Children’s Food Picture Book achieved a similar level of accuracy as the parents’ assessments. One limitation, however, is the low participation rate of the children, despite the relatively large number of children on whom we had food data for. Having more preschools involved would have been advantageous in terms of giving more power to the analyses. Another limitation is that the vegetable consumption variable did not include vegetables in main dishes, but in Finnish preschools, vegetables are mainly eaten separately as salad or raw vegetables. We are aware that if vegetables in main dishes would have been included, the intake of vegetables might have been a bit higher. It should also be noted that the dichotomous fruit intake variable (eaters vs. non-eaters) might rather describe the supply of fruit at preschool and not the children’s willingness to eat it, and thus the results on fruit consumption should be interpreted with caution. Fruit was served on average 2.5 times a week (data not shown), and it is therefore possible that none was served on the two days on which records were kept for some children. Another limitation is that, given the lack of ready-made questionnaires that suited our purposes, we had to formulate the questions we used to assess the preschool-level factors largely by ourselves, and they have not been validated.

## 5. Conclusions

We examined the extent to which preschool-level factors other than food availability and mealtime practices are associated with the intake of vegetables, fruit, and fibre among three- to six-year-old Finnish preschoolers. Written food policies and manager-perceived cooperation-related challenges with the catering service were associated with the children’s dietary intake at preschool. These findings demonstrate that preschool factors not directly related to food availability or feeding practices can still be associated with dietary intake, and these factors could be relevant in the promotion of healthy food intake at preschools. There is clearly a need for more studies focusing on such issues.

## Figures and Tables

**Table 1:** Description of the preschool-level variables *.

Variable	Questions/Statements	Response Options	Categorization
**Policy factors**			
Written food policies	Does the preschool have its own or municipal/national policies related to the following: a. Members of staff encourage children to eat fruit and vegetables b. Using food as a reward or punishment c. Planned food education for the children d. Staff training on children’s nutrition e. Family guidance on children’s nutrition f. Food served on festive days g. Food served on birthdays h. Snacks children bring from home i. Products sold for fund-raising j. Special diets k. Ethical and religious diets l. Target portion sizes for vegetables m. Tasting rules n. Thirst-quenching drinks o. Taking children’s individual preferences into account p. Staff taking meals with the children q. Staff eating food bought from home in the presence of the children r. Where the staff had their coffee break	a. No policies b. Oral policy c. Own written policy d. Municipal or national policy	1 point for each written policy (c or d)
**Sociocultural factors**			
Food education	1. Has the preschool had in-service training for its personnel on child nutrition during the last 2 years?	a. No b. Once c. Twice or more	a = 0 points, b or c = 1 point
	2. Has the preschool had theme weeks about nutrition/food education during the last 2 years?	a. No b. Once c. Twice or more	a = 0 points, b or c = 1 point
	3. Is the Sapere method familiar to you?	a. The method has been used in the preschool b. The method is currently being used in the preschool c. The method is familiar, but it has not been used in the preschool d. The method is not familiar	a or b = 1 point, c or d = 0 points
Cooperation challenges with the catering service	Is the following a barrier to healthy nutrition? Lack of cooperation with the catering service.	Yes/no	Yes = 1 point No = 0 points
	Is the following a barrier to the promotion of healthy nutrition/food education in your preschool? Limitations of the catering service or food supplier.	Yes/no	Yes = 1 point No = 0 points
	How would you describe the communication between the early educators and the catering service staff?	a. Fluent b. There are some challenges.	a = 0 points b = 1 point
Lack of resources as a barrier to healthy nutrition	Is the following a barrier to healthy nutrition? A. Lack of planning time. B. Lack of materials. C. Lack of staff	Yes/no	1 point for each barrier (scale 0–3)
**Manager-related psychosocial factors**			
Concern about children’s fruit and vegetable consumption	To what extent do you think the following matters are generally a problem among 3 to 6-year-old children? A. Low intake of vegetables and B. Low intake of fruit and berries	1 not a problem 2 hardly a problem 3 a problem to some extent 4 quite a big problem 5 a very big problem	Sum of a and b (scale 2–10)
Perceived influence over fruit and vegetable supply	Can the manager influence the supply of fruit and vegetables for different meals?	Yes/no (separate questions for each meal)	Yes to all questions vs. no to all questions
**Physical factors**			
Kitchen type	What type of kitchen facilities do you have in your preschool?	a. Cooking b. Distribution c. Heating d. Other, what? e. There is no kitchen	a and c combined vs. all others

* The questionnaire was available in Finnish and Swedish. The authors did the English translation.

**Table 2:** Descriptive results on the preschool-level factors.

**Policy factors**	
Number of written food policies (scale 0–18)	%
4 or less points	35
5–9 points	36
10 or more points	29
**Sociocultural factors**	
Food education (scale 0–3)	
0 points	45
1 point	34
2 or 3 points	22
Perceived cooperation challenges with the catering service (scale 0–3)	
No challenges	54
1 challenge	24
2 or 3 challenges	22
Lack of resources as a barrier to healthy nutrition	
no barriers	81
1–3 barriers	19
**Manager-related psychosocial factors**	
Concern about children’s fruit and vegetable consumption (scale 2–10)	
5 or less points	19
6 points	44
7 or more points	36
Perceived influence over fruit and vegetable supply	
yes	19
no	81
**Physical factors**	
Kitchen type	
Cooking or heating kitchen	37
Other	63

**Table 3:** Characteristics of the children and of the preschool managers.

	% or Mean (SD)
**Child-level characteristics (*n* = 586)**	
Age, years	4.7 (0.9)
Gender, girls	47%
**Preschool manager’s characteristics (*n* = 53)**	
Gender, women	100%
Age, years	48.4 (7.7)
Educational level	
Bachelor of educational science/Early education teacher	60%
Other	40%
Work experience as a manager, years	13.7 (11.8)

**Table 4:** Children’s dietary intake at preschool.

Children’s Dietary Intake at Preschool (Breakfast, Lunch, and Afternoon Snack) (*n* = 586)	
Energy (kJ)	3229 (910)
Fibre g	9.4 (3.1)
Fibre density g/MJ	3.0 (0.8)
Vegetables (g), raw and cooked	38.5 (28.3)
Fruit consumption, proportion of fruit eaters	63%

**Table 5:** Linear and logistic multi-level regression analyses of preschool-level factors and children’s fruit and vegetable consumption, and fibre intake at preschool.

		Fruit Consumption (Yes vs. No) ^a^						Vegetable Consumption (g/day, Square–Root Modified) ^b^						Fiber Density (g/MJ) ^b^					
		Model 1			Model 2			Model 1			Model 2			Model 1			Model 2		
**Policy factors**		OR	95% CI	*p*	OR	95% CI	*p*	beta	95% CI	*p*	beta	95% CI	*p*	beta	95% CI	*p*	beta	95% CI	*p*
Written food policies	1st tertile (0–4)	1			1			0			0			0			0		
	2nd tertile (5–9)	1.18	(0.40–3.51)	0.76	1.19	(0.48–2.94)	0.70	0.57	(−0.28–1.41)	0.19	0.70	(−0.02–1.41)	0.06	−0.07	(−0.30–0.15)	0.53	−0.02	(−0.21–0.16)	0.82
	3rd tertile (10–18)	1.47	(0.36–5.89)	0.59	0.44	(0.10–1.91)	0.27	0.73	(0.00–1.45)	0.05	0.89	(0.20–1.58)	0.01	0.03	(−0.19–0.24)	0.80	0.24	(0.02–0.46)	0.03
**Sociocultural factors**																			
Food education	Low (0 points)	1			1			0			0			0			0		
	Medium (1 point)	0.75	(0.24–2.31)	0.61	0.62	(0.25–1.52)	0.30	0.20	(−0.48–0.89)	0.56	−0.09	(−0.71–0.53)	0.77	0.05	(−0.16–0.25)	0.66	−0.03	(−0.23–0.17)	0.77
	High (2 or 3 points)	0.70	(0.20–2.41)	0.58	1.44	(0.45–4.58)	0.53	0.10	(−0.88–1.07)	0.84	0.00	(−1.10–1.10)	0.99	0.00	(−0.24–0.24)	0.99	−0.11	(−0.30–0.09)	0.29
Cooperation challenges with the catering service	0	1			1			0			0			0			0		
	1	0.70	(0.23–2.10)	0.53	1.24	(0.50–3.11)	0.64	−0.41	(−1.20–0.39)	0.32	−0.15	(−0.87–0.57)	0.69	−0.02	(−0.26–0.22)	0.87	0.05	(−0.17–0.27)	0.64
	2–3	0.28	(0.10–0.83)	0.02	0.28	(0.11–0.76)	0.01	0.32	(−0.35–0.99)	0.35	0.48	(–0.23–1.18)	0.18	0.15	(−0.04–0.34)	0.12	0.22	(0.03–0.42)	0.03
Lack of resources as a barrier to healthy nutrition	No barriers	1			1			0			0			0			0		
	1–3 barriers	0.24	(0.07–0.82)	0.02	0.44	(0.17–1.13)	0.09	−0.13	(−1.21–0.95)	0.82	0.13	(−0.97–1.22)	0.82	−0.09	(−0.34–0.16)	0.47	−0.06	(−0.31–0.19)	0.65
**Manager–related psychosocial factors**																			
Concern about fruit and vegetable consumption	Low	1			1			0			0			0			0		
	Medium	0.45	(0.14–1.44)	0.18	0.37	(0.12–1.19)	0.10	−0.91	(−0.21–0.49)	0.55	−0.47	(−1.17–0.24)	0.20	0.01	(−0.20–0.22)	0.91	−0.12	(−0.35–0.10)	0.29
	High	1.48	(0.47–4.63)	0.5	1.05	(0.29–3.82)	0.94	−0.28	(−1.06–0.49)	0.47	−0.13	(−1.04–0.78)	0.78	−0.14	(−0.36–0.09)	0.23	−0.18	(−0.42–0.06)	0.14
Perceived influence over fruit and vegetable supply	No	1			1			0			0			0			0		
	Yes	2.95	(0.87–10.00)	0.08	1.83	(0.54–6.16)	0.33	1.01	(0.08–1.95)	0.03	0.68	(−0.55–1.91)	0.28	0.12	(−0.17–0.40)	0.42	−0.08	(−0.38–0.22)	0.6
**Physical factors**																			
Kitchen type	Other	1.00			1			0			0			0			0		
	Cooking/heating	0.29	(0.11–0.76)	0.01	0.58	(0.24–1.44)	0.24	0.04	(−0.69–0.77)	0.91	0.33	(−0.68–1.34)	0.52	0.13	(−0.04–0.31)	0.14	0.17	(−0.04–0.38)	0.12

Model 1: no adjustments; Model 2: adjusted for child’s age, gender and municipality; ^a^ logistic regression; ^b^ linear regression.
